# Author Correction: Reactive astrocytes function as phagocytes after brain ischemia via ABCA1-mediated pathway

**DOI:** 10.1038/s41467-017-01594-1

**Published:** 2017-11-14

**Authors:** Yosuke M. Morizawa, Yuri Hirayama, Nobuhiko Ohno, Shinsuke Shibata, Eiji Shigetomi, Yang Sui, Junichi Nabekura, Koichi Sato, Fumikazu Okajima, Hirohide Takebayashi, Hideyuki Okano, Schuichi Koizumi

**Affiliations:** 1 Department of Neuropharmacology, Interdisciplinary Graduate School of Medicine, University of Yamanashi, Chuo, Yamanashi 409-3898 Japan; 2 Department of Super-network Brain Physiology, Graduate School of Life Science, Tohoku University, Sendai, Miyagi 980-8575 Japan; 3 Division of Neurobiology and Bioinformatics, National Institute for Physiological Sciences, Okazaki, Aichi 444-8585 Japan; 4 Department of Physiology and Electron Microscope Laboratory, Keio University School of Medicine, Shinjuku, Tokyo 160-8582 Japan; 5 Division of Homeostatic Development, National Institute for Physiological Sciences, Okazaki, Aichi 444-8585 Japan; 6 Department of Physiological Sciences, The Graduate School for Advanced Study, Hayama, Kanagawa 240-0193 Japan; 7 Laboratory of Signal Transduction, Institute for Molecular and Cellular Regulation, Gunma University, Maebashi, Gunma 371-8512 Japan; 8 Division of Neurobiology and Anatomy, Graduate School of Medical and Dental Sciences, Niigata University, Niigata, Niigata 951-8510 Japan

*Nature Communications* **8**: 28 doi:10.1038/s41467-017-00037-1; Article pubilshed online: 22 June 2017

The original version of this Article contained an error in the spelling of the author Nobuhiko Ohno, which was incorrectly given as Noubuhiko Ohno. This has now been corrected in both the PDF and HTML versions of the Article.

