# Supplementary material for: Reactive astrocytes function as phagocytes after brain ischemia via ABCA1-mediated pathway
Source: Nat Commun. 2017 Jun 22;8:28. doi: 10.1038/s41467-017-00037-1 (PMC5481424; doi:10.1038/s41467-017-00037-1)
Supplement: Supplementary file 1 — Supplementary Information [file 41467_2017_37_MOESM1_ESM.pdf]

File name: Supplementary Information
Description: Supplementary Figures

File name: Peer Review File
Description:

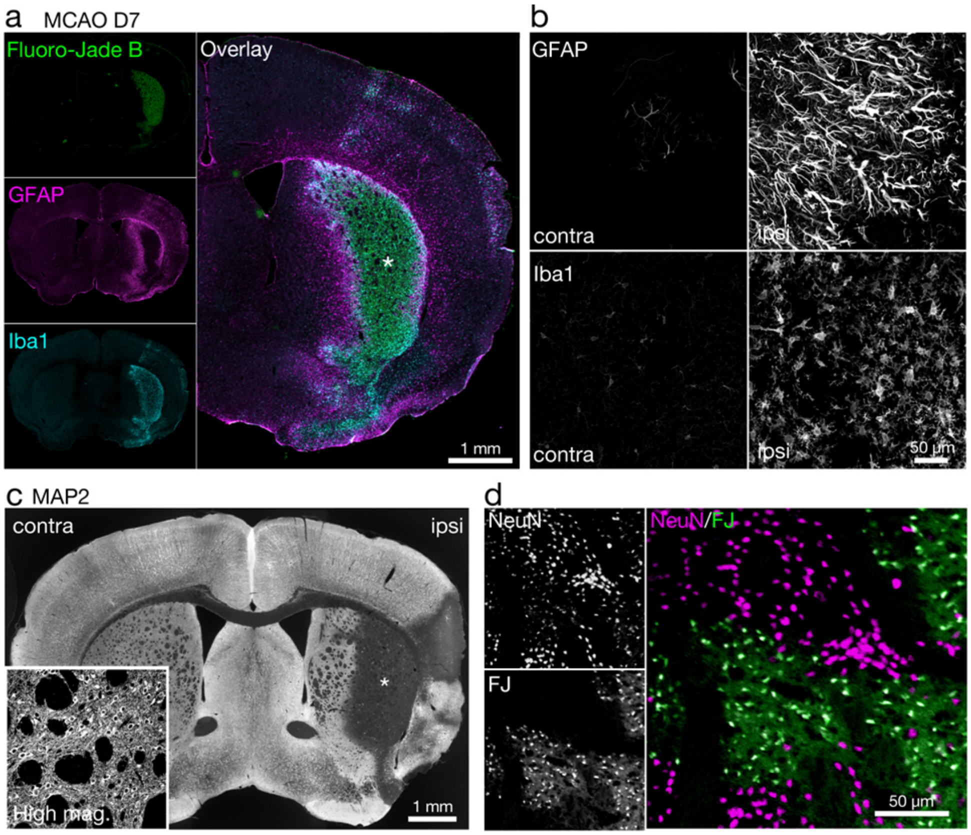

Supplementary Figure 1. Transient ischemic injury induces neuronal degeneration and reactive gliosis.

(a) Representative images of Fluoro-Jade B-labelled (FJ, green) and GFAP- (magenta) and Iba1 (cyan)-immunostained brain sections 7 days after MCAO. FJ labelled degenerating neurons in the dorsolateral striatum on the ipsilateral side. **(b)** High-magnification images of GFAP-immunostained astrocytes (upper) and Iba1-immunostained immune cells (lower) in the penumbra area of the ipsilateral striatum (ipsi, right) and the ischemic core area of ipsilateral striatum (ipsi, right), respectively, and in the contralateral striatum 7 days after MCAO (contra, left). **(c)** Representative images of MAP2 immunoreactivity 7 days after MCAO. MAP2 immunoreactivity is not observed in the ipsilateral striatum, as shown by FJ labelling. **(d)** Representative images of FJ (green)-labelled and NeuN (magenta)-immunostained brain sections in the ipsilateral cortex 5 days after MCAO. FJ+ large degenerating neurons were 100% NeuN+ (n=241 cell, 3 mice).

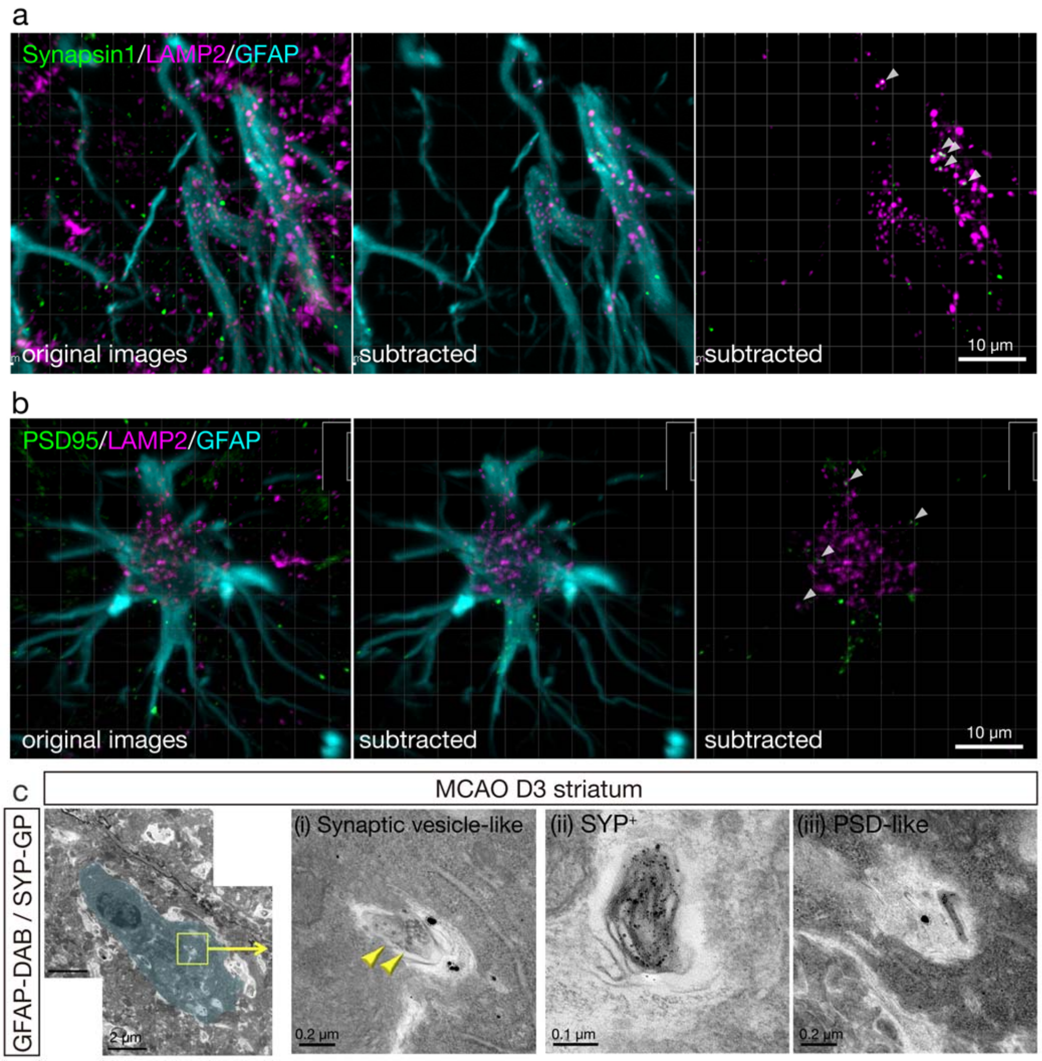

Supplementary Figure 2. Astrocytes engage in synapse elimination after transient ischemic injury.

(a, b) Three-dimensional surface rendering before (original image, left) and after (subtracted, middle and right) by subtracting Synapsin1 (a) or PSD95 (b) outside of the astrocyte (GFAP). Representative images show reactive astrocytes (GFAP, cyan) and engulfed synapses (Synapsin1^{+} pre-synapses (a) or PSD95^{+} post-synapses (b), green) co-localized with LAMP2^{+} lysosome (magenta) in the ipsilateral striatum 7 days after MCAO. Arrowheads indicate colocalisation of synaptic markers and lysosomes. Fifty-two (a) and 45 (b) images per z stack (0.38 μm step). **(c)** Synaptic vesicle structure is visible inside of the GFAP-DAB^{+} astrocyte **(i)**. Synaptophysin-positive (GP) phagocytic inclusion is inside of the GFAP-DAB^{+} astrocyte **(ii)**. PSD-like structure is detected inside of the GFAP-DAB^{+} astrocyte **(iii)**.

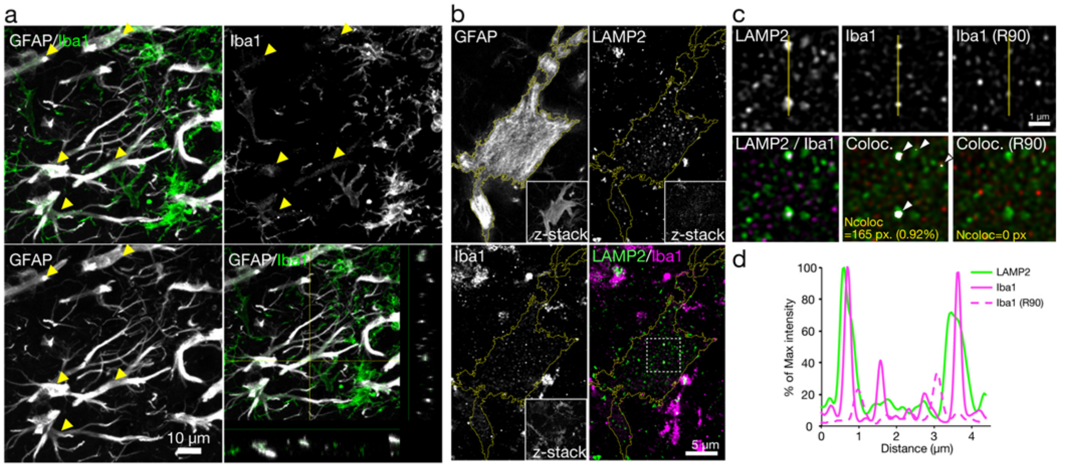

Supplementary Figure 3. Astrocytes engulf $Iba1^+$ fractions after transient ischemic injury. **(a)** Representative images showing Iba1 (green) signals in GFAP (white) cells at 10 days after MCAO. Arrowheads indicate colocalisation. Thirty-six images per z stack (0.38 µm step). **(b)** Representative images show the $Iba1^+$ fraction (magenta) and lysosome (LAMP2, green) in reactive astrocytes (GFAP, yellow ROI) in the ipsilateral striatum at 7 days after MCAO. Large panels show single plane images and small insets show z-stack images. Twenty-eight images per z stack (0.2 µm step). **(c)** High magnification images of box with dotted line in LAMP2/Iba1 (b) show Iba1 signals colocalize with LAMP2 signals (c, arrowheads, Coloc). A 90-degree rotation of Iba1 panel (R90) shows no colocalisation with LAMP2 signals (c, right, Coloc (R90)). (d) Line profiles show immunofluorescence intensities of Iba1, and LAMP2 along the line (c, yellow).

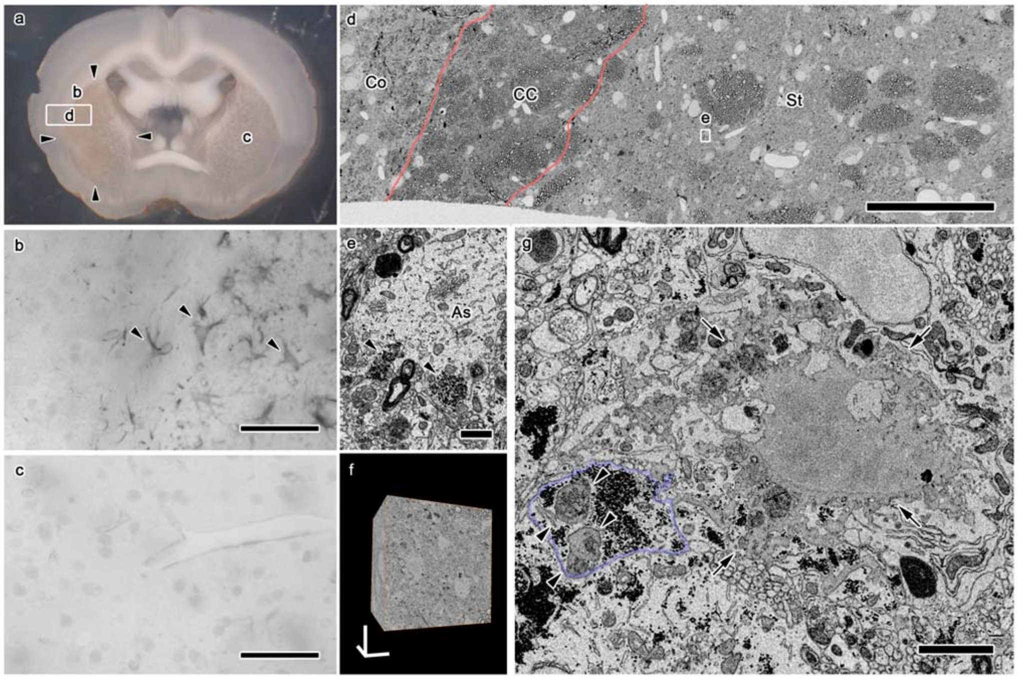

Supplementary Figure 4. Sampling and image acquisition procedures of serial electron microscopic images with serial block face scanning electron microscopy (SBF-SEM).

(a, b) Immunohistochemical staining for GFAP shows dense DAB deposits (a, arrowheads) around the ipsi-lateral striatum, in which reactive astrocytes immunopositive for GFAP (b, arrowheads) are observed in penumbra regions at a higher magnification. **(c)** GFAP-positive astrocytes are not observed in the contra-lateral striatum. **(d-f)** In SBF-SEM, striatum was identified at low magnification (d, St), and serial electron microscopic images (e) were acquired and reconstructed (f). Glycogen granules appear as electron dense granules (e, arrowheads) and are frequently found in the cytoplasm of astrocytes (As). **(g)** In the ischemic lesion, large cellular debris with a complex contour (arrows) and small debris (arrowheads) was engulfed by cytoplasm (blue). CC: corpus callosum, Co: cortex. Bars: 100 μm (b-d), 2 μm (e, g) or 10 μm (f).

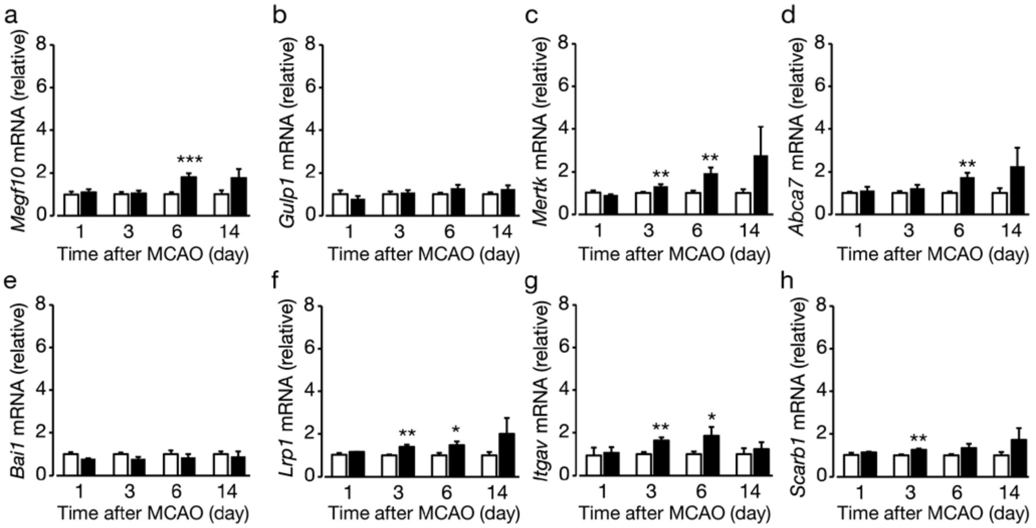

Supplementary Figure 5. Gene expression analysis of phagocytosis-related molecules after transient ischemic injury.

(a–h) Real-time PCR analysis of phagocytosis-related molecules extracted from the ipsilateral or contralateral striatum after MCAO. Values represent the relative ratio of respective mRNAs (normalized to GAPDH mRNA levels) to the contralateral striatum after MCAO (D1: n = 4, 3, 4, 3, 3, 4, 4, 4; D3: n = 8, 4, 8, 4, 4, 8, 8, 8; D6: n = 8, 7, 8, 10, 8, 8, 8, 8; D14: n = 6, 3, 6, 6, 6, 6, 6, 6 (in alphabetical order), *P < 0.05, **P < 0.01, ***P < 0.001 vs. contra (corresponding day), unpaired t-test).

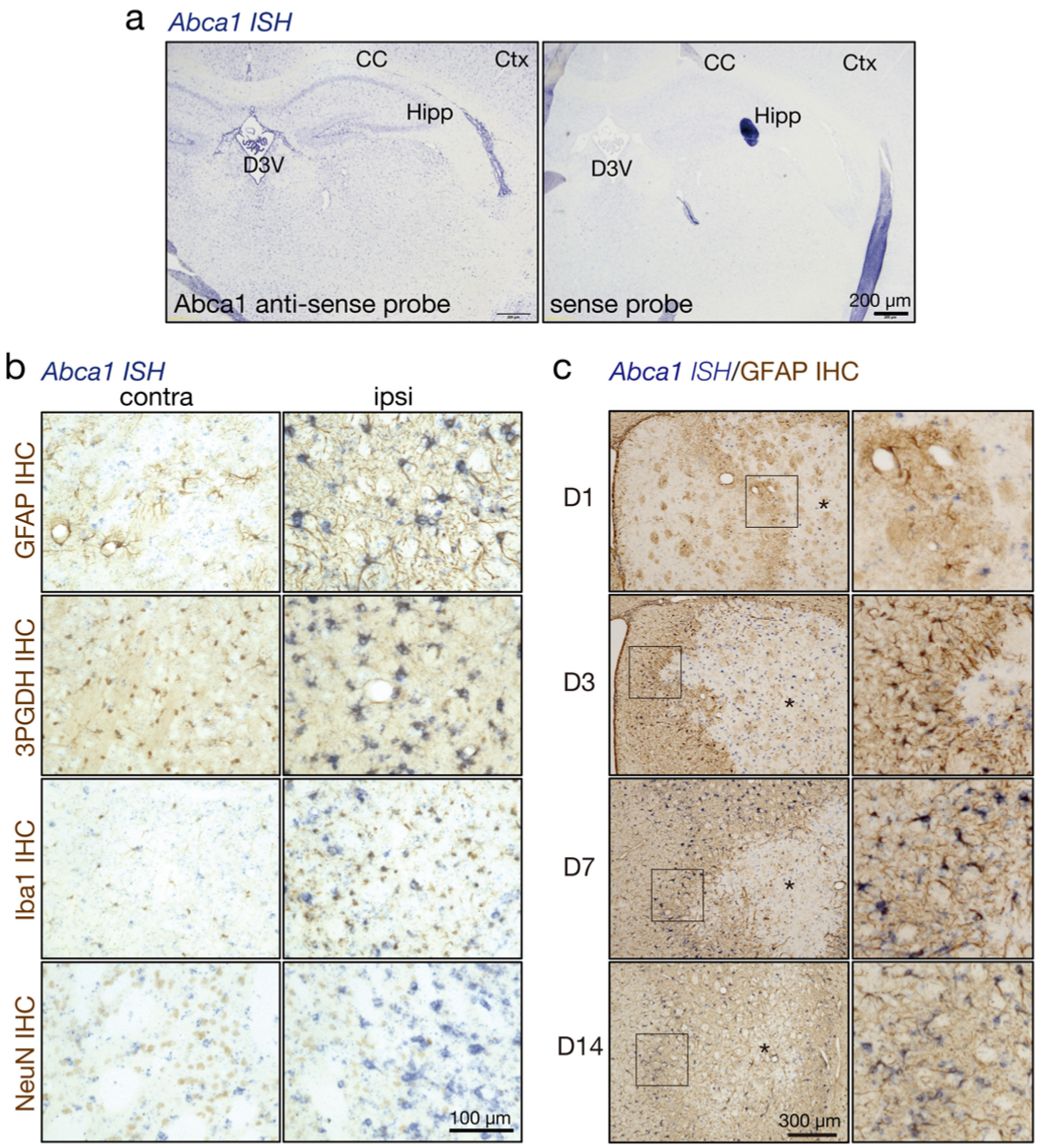

Supplementary Figure 6. Abca1 mRNA upregulation in reactive astrocytes after transient ischemic injury.

(a) *In situ* hybridization of intact brain tissue in the presence of Abca1 antisense and sense riboprobes. **(b)** Representative images show *Abca1* ISH signals and GFAP, 3PGDH, Iba1, or NeuN IHC signals in the contralateral and ipsilateral striatum 7 days after MCAO. **(c)** Temporal changes in Abca1 ISH signals in the ischemic penumbra area.

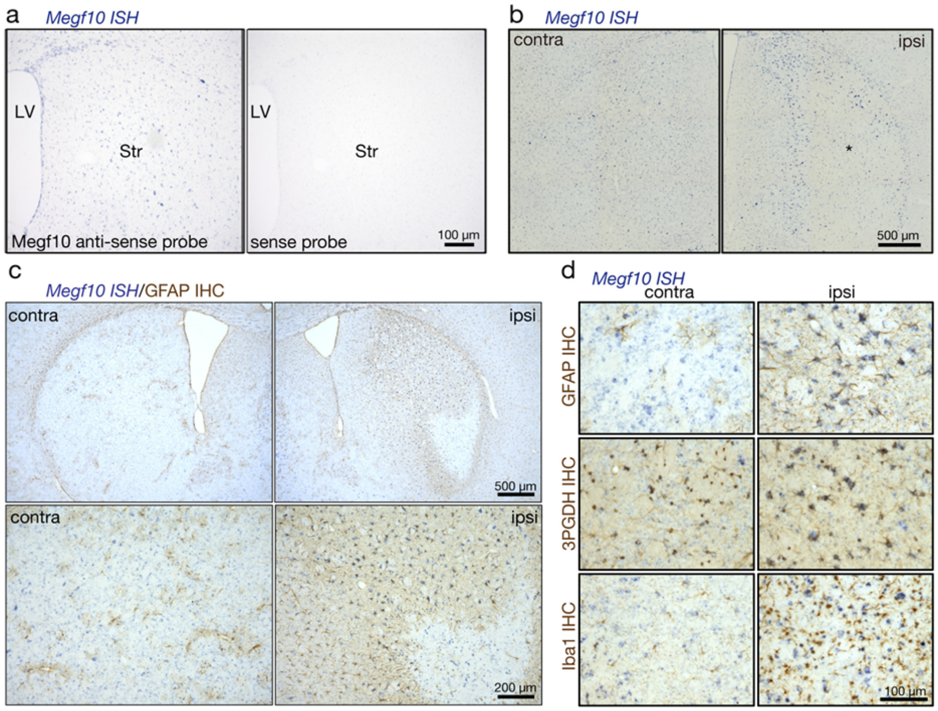

Supplementary Figure 7. Megf10 mRNA upregulation in reactive astrocytes after transient ischemic injury.
(a) *In situ* hybridization of intact brain tissue in the presence of Megf10 antisense and sense riboprobes. **(b)** *In situ* hybridization (ISH) analysis of Megf10 mRNA 7 days after MCAO. Megf10 ISH signals (purple) are upregulated in the ischemic penumbra. **(c)** Representative images show Megf10 ISH signals that colocalize with GFAP immunoreactivity (DAB: brown). **(d)** Representative images show Megf10 ISH signals and GFAP, 3PGDH, or Iba1 IHC signals in the contralateral and ipsilateral striatum 7 days after MCAO.

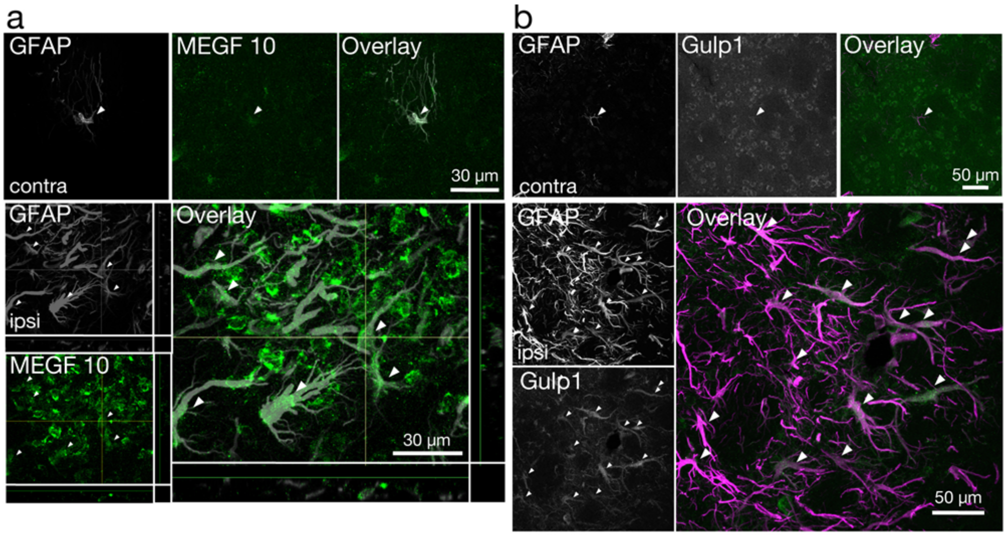

Supplementary Figure 8. Transient ischemic injury induces MEGF10 and GULP1 upregulation in reactive astrocytes. Representative images show immunohistochemical staining for MEGF10 **(a)** and Gulp1 **(b)** in the striatum 7 days after MCAO. Arrowheads indicate $GFAP^{+}$ astrocytes. Twenty-eight **(a)** and 34 **(b)** images per z stack (0.38 µm step).

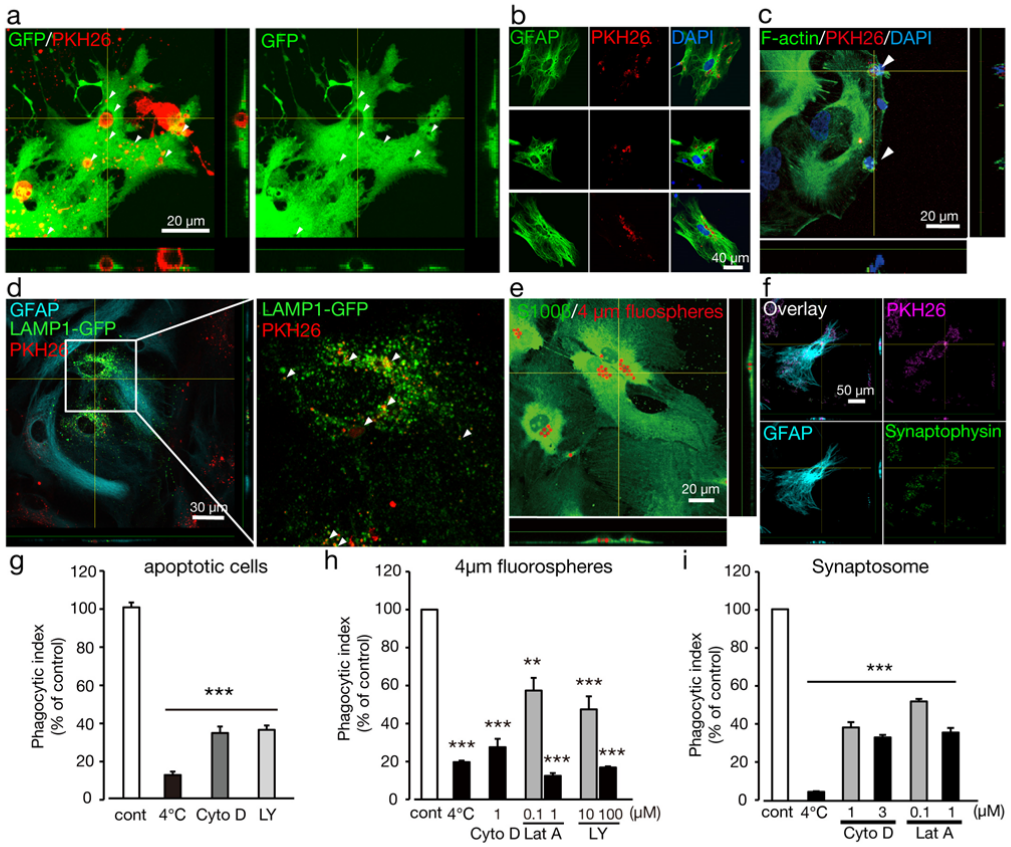

Supplementary Figure 9. Astrocytes engulf neuronal debris, synthetic targets, and synapses *in vitro.*

(a) Cultured astrocytes expressing GFP were incubated with PKH26 (magenta)-labelled apoptotic neuronal cell debris. Images are compiled from orthogonal sections showing apoptotic neuronal debris inside of the astrocyte. Arrowheads indicate engulfed debris (3 h after incubation). Eleven images per z stack (1 µm step)**. (b)** Representative images of astrocytes engulfing neuronal debris (6 h). **(c)** Representative images of actin polymerization (phalloidine-FITC) during engulfment (1 h). Arrowheads indicate phagocytic cup structures. Eleven images per z stack (1.5 µm step)**. (d)** Representative image of LAMP1 recruitment to phagolysosomes (arrowheads) in astrocytes expressing LAMP1-GFP and incubated with apoptotic neurons (12 h). Twenty-seven images per z stack (0.39 µm step). **(e)** Images showing 4-µm beads inside of astrocytes (S100β) (12 h). Thirty images per z stack (0.5 µm step). **(f)** Representative images of astrocytes (GFAP) engulfing synaptosomes (PKH26). Synaptophysin-positive synaptosomal fractions inside the astrocyte cytoplasm (1 h). Twenty-two images per z stack (0.47 µm step). **(g)** Phagocytosis of apoptotic cells in the presence or absence of drugs, presented as a phagocytic index. Astrocytes were pretreated with actin polymerization inhibitor cytochalasin D

(Cyto D) or PI3K inhibitor LY294002 for 15 min prior to addition of PKH26-labeled apoptotic neurons. Mixed cells were incubated for 3–5 h at 37°C or 4°C, and the uptake of targets was assessed by FACS-based phagocytosis assay (n = 8, 4, 4, 4, ****P* < 0.001, unpaired t-test). **(h)** Effects of drugs on astrocytic phagocytosis of 4-μm beads (n =8, 4, 5, 3, 4, 4, 4, ***P* < 0.01, ****P* < 0.001, unpaired t-test). **(i)** Effects of drugs on astrocytic phagocytosis of synaptosomes (n = 4, ****P* < 0.001, unpaired t-test). Values represent means ± SEM.

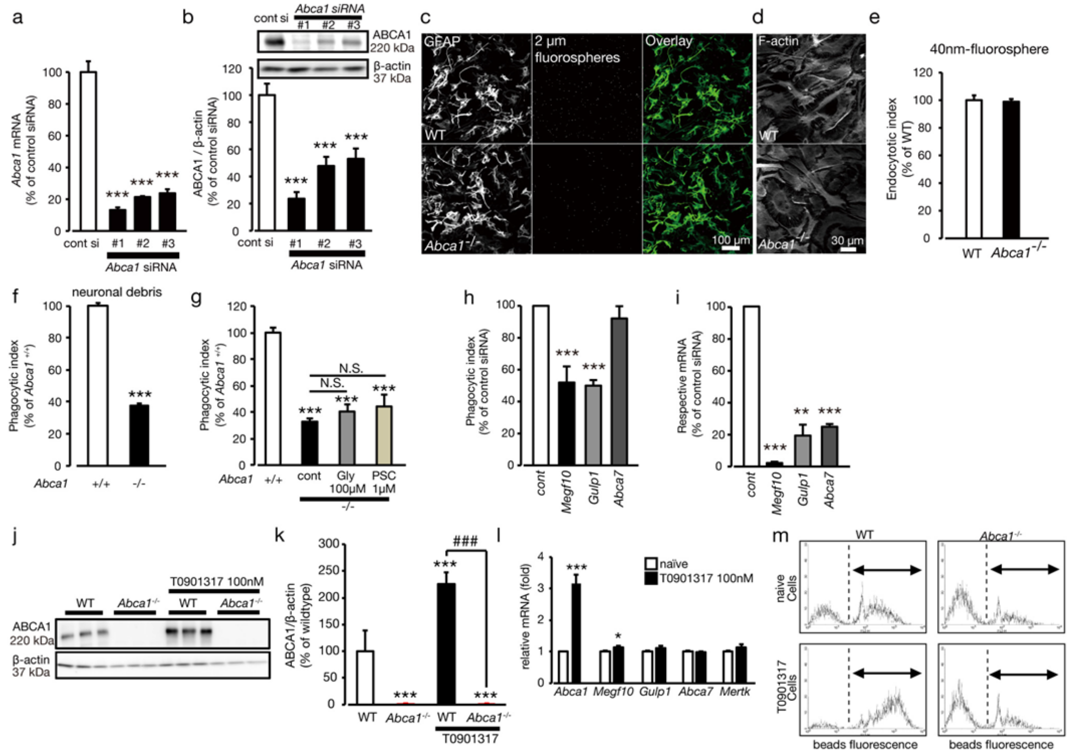

Supplementary Figure 10. Knockdown or knockout of Abca1 expression, in combination with LXR agonist T0901317 treatment, exclusively upregulates *Abca1* expression in astrocytes.

(a, b) Abca1 mRNA expression (a: normalized to GAPDH mRNA (n = 9, 9, 3, 3) and ABCA1 protein (b: normalized to β-actin (right, n = 8, 5, 6, 7)) in astrocytes transfected with control siRNA or Abca1 siRNAs (****P* < 0.001 vs. control siRNA, unpaired t-test). **(c)** Images of WT and ABCA1$^{-/-}$ astrocytes after 1 h of phagocytosis of 2-μm beads. **(d)** Actin-filaments (phalloidine-FITC) of astrocytes from WT or Abca1$^{-/-}$ mice. **(e)** Quantitative analysis of endocytosis of 40-nm beads shows that Abca1$^{-/-}$ astrocytes are not significantly different from WT astrocytes (n = 4, 8, *P* = 0.5061, unpaired t-test). **(f)** Phagocytic activities of neuronal cell debris by astrocytes from Abca1$^{+/+}$ or Abca1$^{-/-}$ mice (n = 4, ****P* < 0.001, unpaired t-test). (g) Phagocytic activities in the presence of ABCA1 inhibitors. Glyburide and PSC833 did not affect engulfment by Abca1-deficient astrocytes (n = 4, ****P* < 0.001 vs. Abca1$^{+/+}$, one-way ANOVA (P < 0.0001) with Tukey's multiple comparison test). (h) Phagocytic activities of astrocytes transfected with control siRNA, Megf10, Gulp1, or Abca7 siRNAs (n =10, 5, 8, 7; ***P < 0.001 vs. control siRNA, unpaired t-test). **(g)** Bar graph shows Megf10, Gulp1, and Abca7 mRNA expression normalized to GAPDH mRNA levels in astrocytes transfected with control siRNAs or Megf10, Gulp1, or Abca7 siRNAs, respectively (n = 3; ***P* < 0.01, ****P* < 0.001, unpaired t-test). **(h)** ABCA1 protein

of astrocytes from WT or Abca1$^{-/-}$ mice with or without T0901317 pretreatment (100 nM, 48 h). **(i)** ABCA1 band intensities are shown in the bar graph (n = 3, ****P* < 0.001 vs. WT naïve, unpaired t-test). **(j)** Real-time PCR analysis of phagocytosis-related molecules extracted from astrocytes with or without T0901317 treatment (100 nM, 48 h). Values represent the relative ratio of respective mRNAs (normalized to GAPDH mRNA levels) to naïve (n = 5, 6; **P* < 0.05, ****P* < 0.001 vs. naïve, unpaired t-test). **(k)** Representative FACS histograms of beads intensity in astrocyte populations after incubating with 2-μm beads for 1 h. T0901317-treated WT astrocytes (lower left) show a clear rightward shift in bead intensity compared with non-treated WT astrocytes (upper left). This shift is absent in Abca1$^{-/-}$ astrocytes. Values represent means ± SEM.

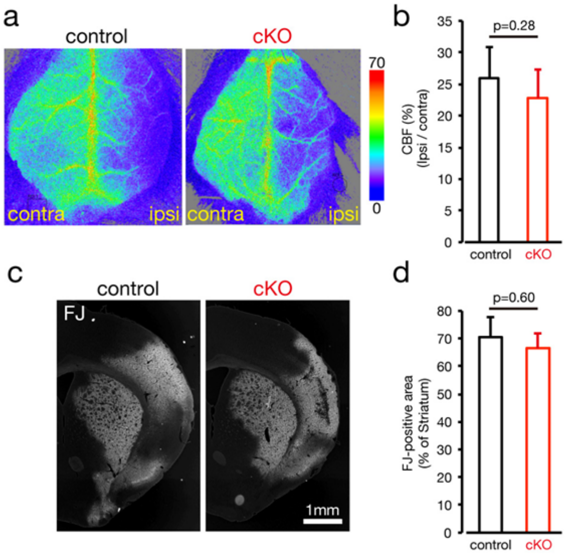

Supplementary Figure 11. CBF during occlusion and brain damage after MCAO in control and ABCA1-cKO mice.

(a, b) CBF reduction in the ipsilateral hemisphere during occlusion was comparable between control and cKO mice (n=6, unpaired t-test). **(c, d)** Macroscopic observations of neuronal degeneration in the striatum were also comparable between control and cKO mice 7 days after MCAO (n= 3, 4, unpaired t-test).

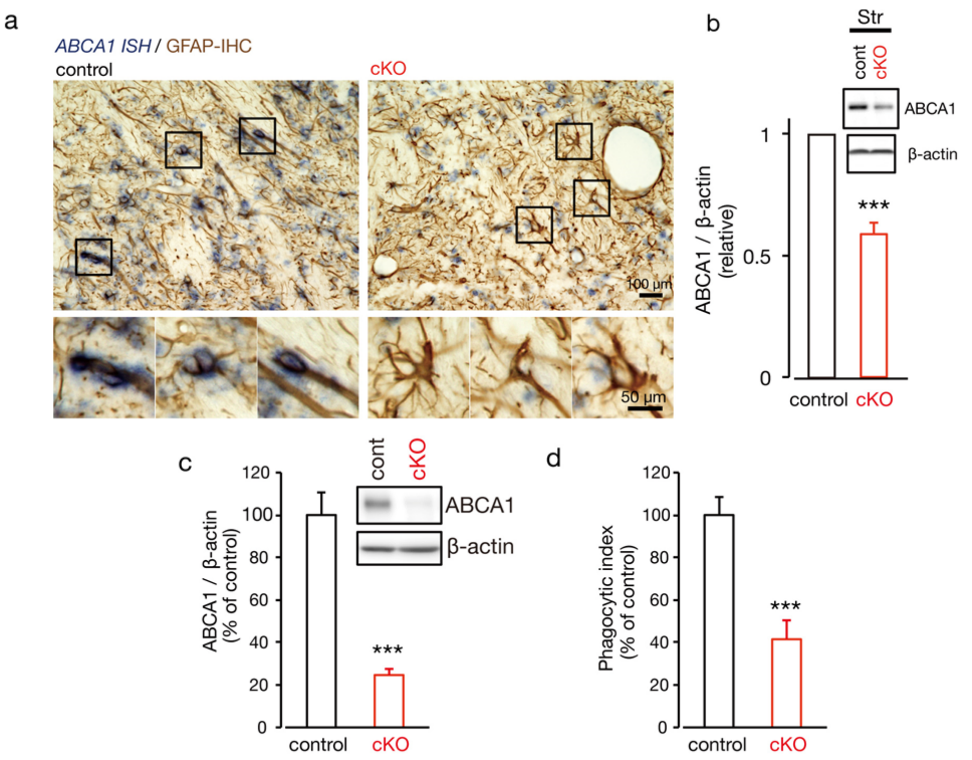

Supplementary Figure 12. Efficiency of Abca1 deletion in ABCA1-cKO mice.
(a) Representative images show *Abca1* ISH signals and GFAP IHC in the ipsilateral striatum in control and cKO mice 7 days after MCAO. High magnification images show lack of Abca1 upregulation in reactive astrocytes from cKO mice. **(b)** Loss of ABCA1 protein was analysed in intact striatum (Str) of cKO mice (n=3, ***P<0.001, unpaired t-test). **(c)** Phagocytic activities of cultured astrocytes from littermate controls and cKO mice (n =3; ***P < 0.001 vs. littermate control, unpaired t-test). **(d)** ABCA1 protein in cultured astrocytes from littermate controls and cKO mice (n =3; ***P < 0.001 vs. littermate control, unpaired t-test).

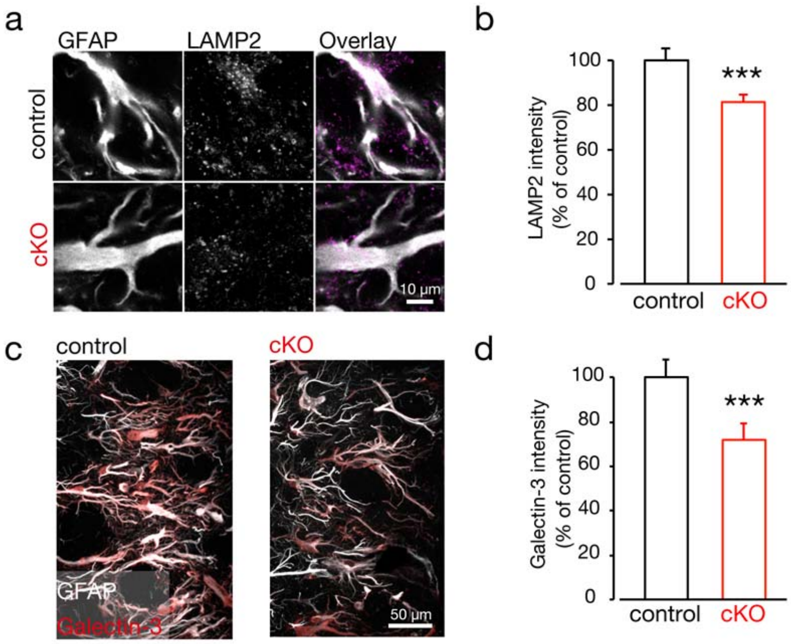

Supplementary Figure 13. ABCA1-deficient astrocytes express fewer phagocytic markers.

(a) Representative images of LAMP2 and GFAP IHC in the ischemic penumbra of control and cKO mice. **(b)** Immunoreactivity of LAMP2 in GFAP-positive cells from cKO mice was significantly lower than in control mice (n =6; *** P < 0.001 vs. littermate control, unpaired t-test). **(c)** Representative images of Galectin-3 and GFAP IHC in the ischemic penumbra of control and cKO mice. **(d)** Immunoreactivity of Galectin-3 in GFAP-positive cells from cKO mice was significantly lower than in control mice (n =6; *** P < 0.001 vs. littermate control, unpaired t-test). Ten images per z stack (2.0 µm step).

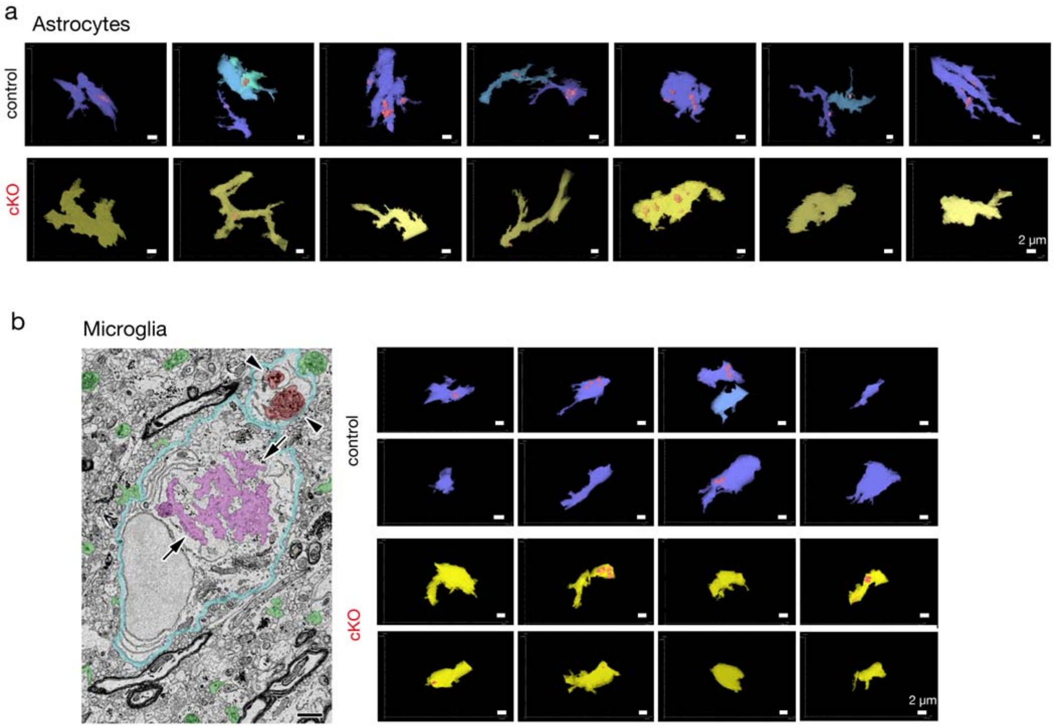

Supplementary Figure 14. List of reconstruction images of astrocytes and microglia in control and cKO mice.

(a) Skeletonizations of astrocytic processes acquired from the ischemic penumbra of control (blue) and ABCA1-cKO (cKO; yellow) mice. Engulfed debris is shown in red. **(b)** Macrophages engulf large and small cellular debris in the ischemic penumbra of both control and cKO mice. In the ischemic penumbra of cKO mice containing cellular debris (green), macrophages (light blue) engulf both large (pink, arrows) and small (red, arrowheads) debris. Bar: 1 μm. Skeletonization of microglia processes acquired from the ischemic penumbra of control (blue) and cKO (yellow) mice. Engulfed debris is shown in red.

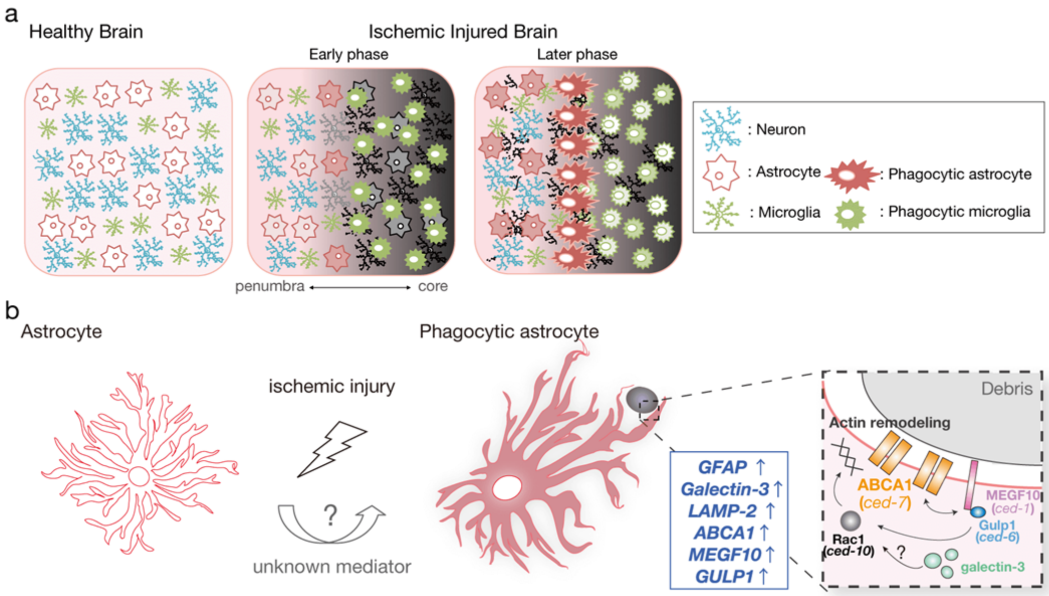

Supplementary Figure 15. Schematics of the spatiotemporal profiles of phagocytic glia and the molecular mechanisms underlying astrocytic phagocytosis.

(a) Engulfment activity of microglia takes place during early onset within the ischemic core, whereas astrocytes become active during late onset within the ischemic penumbra. **(b)** Astrocytes transform into a phagocytic phenotype following increased expression of ABCA1 and its related molecules following ischemic injury.

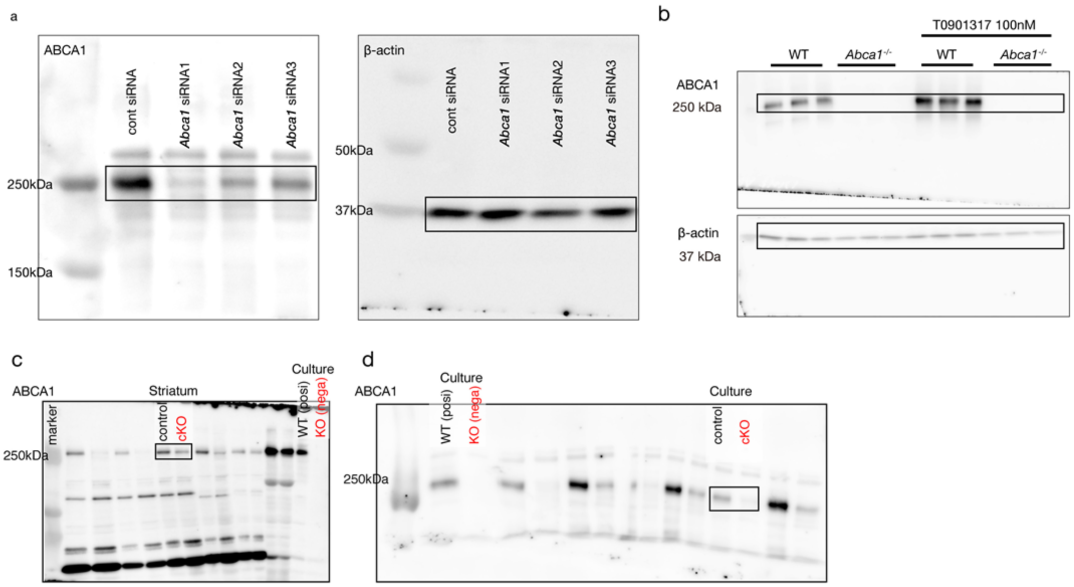

199 **Supplementary Figure 16. (a-d)** Unedited full blots of Supplementary Figure 10b, j, 12b and c,
200 respectively.
